# Effect of carbon-fiber-reinforced polyetheretherketone on stress distribution in a redesigned tumor-type knee prosthesis: a finite element analysis

**DOI:** 10.3389/fbioe.2023.1243936

**Published:** 2023-09-26

**Authors:** Han Wu, Yu Guo, Wei Guo

**Affiliations:** ^1^ Department of Musculoskeletal Tumor, People’s Hospital, Peking University, Beijing, China; ^2^ Beijing Key Laboratory of Musculoskeletal Tumor, Beijing, China

**Keywords:** tumor-type prosthesis, prosthetic reconstruction, carbon-fiber-reinforced polyetheretherketone, finite element analysis, stress distribution

## Abstract

**Background:** Surgery for bone tumors around the knee often involves extensive resection, making the subsequent prosthetic reconstruction challenging. While carbon fiber-reinforced polyetheretherketone (CF-PEEK) has been widely used in orthopedic implants, its application in tumor-type prosthesis is limited. This study aims to evaluate the feasibility of using 30wt% and 60wt% carbon fiber-reinforced polyetheretherketone (CF30-PEEK and CF60-PEEK) as materials for a redesigned tumor-type knee prosthesis through numerical analysis.

**Methods:** A knee joint model based on CT data was created, and the resection and prosthetic reconstruction were simulated. Three finite element models of the prostheses, representing the initial and updated designs with CoCrMo and CFR-PEEK components, were constructed. Loading conditions during standing and squatting were simulated with forces of 700 N and 2800 N, respectively. Finite element analysis was used to analyze the von Mises stress and stability of all components for each prosthesis type.

**Results:** After improvements in both material and design, the new Type 3 prosthesis showed significantly lower overall stress with stress being evenly distributed. Compared with the initial design, the maximum von Mises stress in Type 3 was reduced by 53.9% during standing and 74.2% during squatting. In the standing position, the maximum stress in the CF30-PEEK femoral component decreased by 57.3% compared with the initial design which was composed of CoCrMo, while the stress in the CF60-PEEK cardan shaft remained consistent. In the squatting position, the maximum stress in the femoral component decreased by 81.9%, and the stress in the cardan shaft decreased by 46.5%.

**Conclusion:** The incorporation of CF30-PEEK effectively transmits forces and reduces stress concentration on the femoral component, while CF60-PEEK in the redesigned cardan shaft significantly reduces stress while maintaining stiffness. The redesigned prosthesis effectively conducts loading force and demonstrates favorable biomechanical characteristics, indicating the promising potential of utilizing CF30-PEEK and CF60-PEEK materials for tumor-type knee prostheses. The findings of this study could provide novel insights for the design and development of tumor-type knee prostheses.

## 1 Introduction

Aggressive bone and soft tissue tumors around the knee often require extensive surgical resection, and functional reconstruction with tumor-type knee prosthesis. The limitations of widely used metallic materials in orthopedic prostheses, such as titanium alloys and CoCrMo, have been highlighted in numerous studies. The most common drawbacks are the heavy weight due to high density, and the significant difference in elastic modulus between the metal implants (ranging from 100 to 200 GPa) and human bones (3–20 GPa). The modulus mismatch, also known as the stress shielding effect, can lead to small fractures, aseptic loosening, and ultimately implant failure (Sumner, 2015). Additionally, certain metal ions released by wear particles are toxic to bone and tissue cells, causing inflammation in adjacent tissues (Okazaki and Gotoh, 2005; Scharf et al., 2014; Armstead et al., 2017). Therefore, updating the materials used for prosthetic fabrication is crucial.

Implants that offer suitable mechanical properties, excellent wear resistance, and low cytotoxicity form the basis for successful osteointegration. Carbon/polymer composites have gained significant interest due to their exceptional mechanical properties. Among these composites, carbon-fiber-reinforced polyetheretherketone (CF-PEEK) has been extensively studied in orthopedics over the past few decades, following the successful utilization of pure polyetheretherketone (PEEK) (Theivendran et al., 2021). CF-PEEK provides translucency under X-ray imaging, eliminating scattering effects and enabling the evaluation of early tumor recurrence and precise radiotherapy while minimizing the impact on surrounding soft tissues (Zimel et al., 2015; Nevelsky et al., 2017). CF-PEEK filled with 30wt% short carbon fiber (CF30-PEEK) demonstrates excellent mechanical properties, with a tensile strength (175–209 MPa) and elastic modulus (16–24 GPa) (Bonnheim et al., 2019; Avanzini et al., 2022) significantly lower than those of metals, closely resembling the properties of bone (50–100 MPa and 7–30 GPa) (Morgan et al., 2018). This resemblance effectively avoids stress shielding and subsequent implant loosening or failure (Sha et al., 2009). Additionally, CF-PEEK exhibits favorable wear resistance when articulating against ceramic and metallic materials (Scholes and Unsworth, 2007; Brockett et al., 2016), and its wear particles do not demonstrate significant cytotoxicity (Utzschneider et al., 2010), thereby extending the prosthesis lifespan. To date, CF-PEEK has been investigated in various applications, including spinal cages (Schwendner et al., 2023), fixation systems (Boriani et al., 2018; Cofano et al., 2020), knee joint prostheses (Koh et al., 2019), and intramedullary nails (Vles et al., 2019; Ziran et al., 2020).

The length, arrangement, and weight percentage of carbon fibers filled in CF-PEEK can impact its mechanical properties, providing the opportunity to manipulate them (Liao et al., 2020). While CF-PEEK with 30wt% carbon fiber is reinforced by short carbon fibers, CF-PEEK with 60wt% (CF60-PEEK) contains relatively longer or continuous carbon fibers. CF60-PEEK shares many similarities with CF30-PEEK but exhibits higher stiffness due to the utilization of long carbon fibers (Zhao et al., 2021). The elastic modulus of CF60-PEEK can range from 50 GPa to 150 GPa, depending on the orientation and volume fraction of carbon fibers (Bonnheim et al., 2019). Therefore, CF60-PEEK, possessing light weight and high stiffness, is suitable for fabricating torsion-resistant and load-bearing implants.

Due to the large bone defect, tumor-type knee prostheses are typically heavier than most knee joint implants. Therefore, replacing metallic biomaterials with CF-PEEK holds technological importance for tumor-type prostheses. With a lower density than CoCrMo alloy (7.9–8.5 g/cm^3^) and Ti6Al4V alloy (4.51 g/cm^3^), CF30-PEEK (1.35–1.4 g/cm^3^) can reduce the weight of the prosthesis and alleviate the burden on surrounding tissues, resulting in a considerable improvement in comfort. Despite the increasing use of CF-PEEK in orthopedic trauma and spinal instrumentation, there is limited published research on tumor-type knee prostheses composed of CF-PEEK. Finite Element Analysis is an essential method for simulating stress distribution and exploring mechanical properties when designing a prosthesis (Yao et al., 2021; Zhu et al., 2021). In our previous study, we introduced an originally designed micro-motion tumor-type knee prosthesis and conducted Finite Element Analysis. However, the initial prosthesis yielded unsatisfactory results during subsequent tests. In this study, we have renewed the design and provided a numerical study of a novel tumor-type prosthesis composed of CF-PEEK. This study aims to explore the feasibility of using CF30-PEEK and CF60-PEEK as replacements for CoCrMo in tumor-type knee prostheses, and three different Finite Element models were established to analyze the von Mises stress of each component. The results of this study are expected to provide insights into the development of tumor-type knee prostheses.

## 2 Materials and methods

### 2.1 Updates in prosthesis design

**FIGURE 1:**
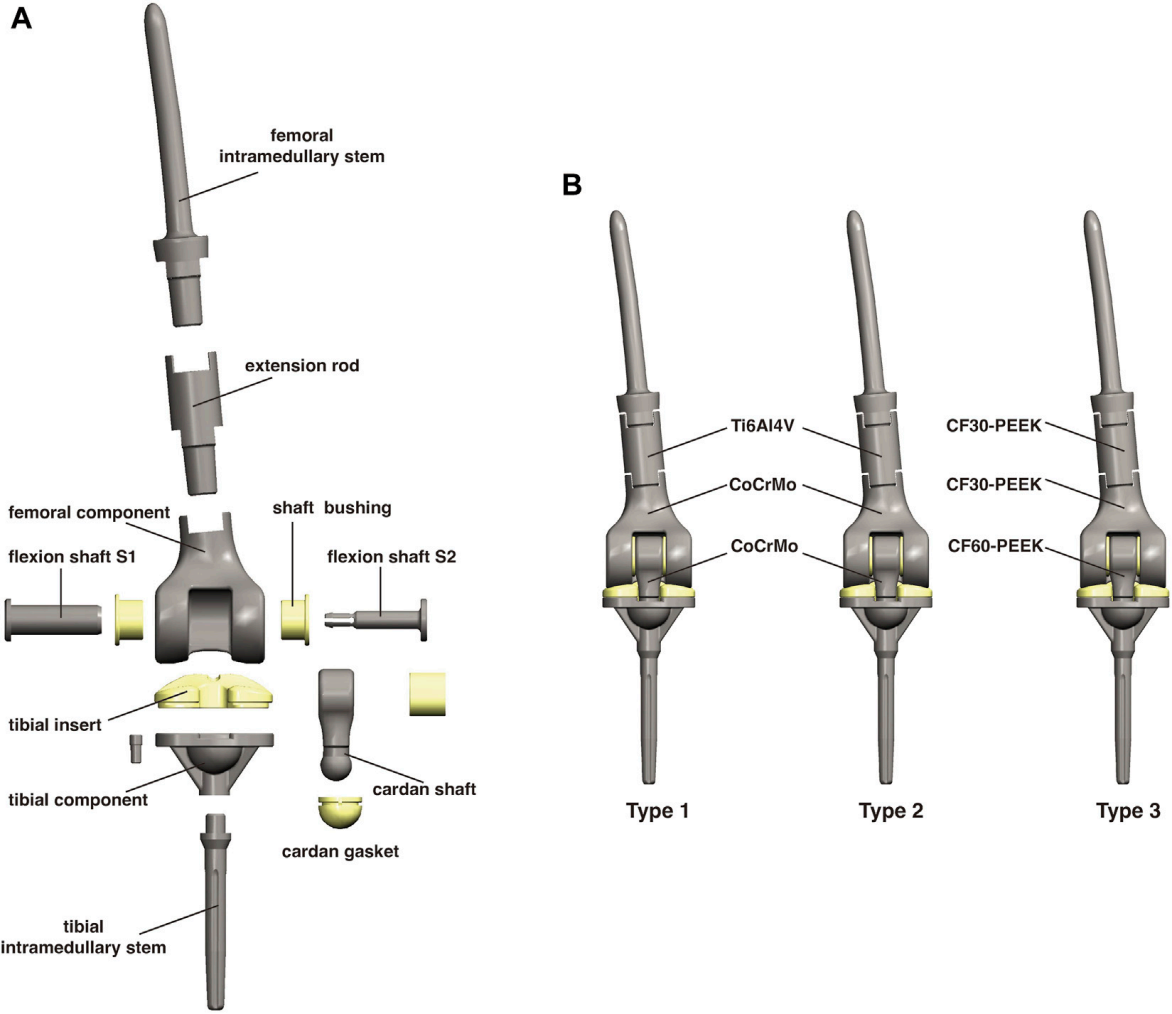
**(A)** A schematic diagram of the components of this micro-motion tumor-type knee prosthesis. **(B)** Differences in the materials among the three types of prostheses.

Three tumor-type knee prostheses, referred to as Type 1, Type 2, and Type 3, were evaluated in this study. Type 1 represents the initial design, consisting of intramedullary stems for the femur and tibia medullary cavity, an extension rod, a distal femoral component, two flexion shafts with two shaft bushings, a cardan shaft with a cardan gasket, a tibial insert, and a proximal tibial component (Figure 1A). The cardan shaft not only links the femur and tibia but also enables controlled micro-motions within the knee joint. This design preserves knee joint flexibility for multidirectional movement while maintaining necessary constraint. However, subsequent test results of Type 1 indicated a possibility of dislocation and the need for further adjustments, leading to the development of Type 2 and Type 3 prostheses.

To reduce the risk of dislocation, the diameter of the distal end of the cardan shaft was enlarged in Type 2 and Type 3, making it wider than the gap of the tibial insert. As a result, the cardan gasket was removed to fit into the groove of the tibial component. Additionally, the neck of the cardan shaft was lengthened, and the PE tibial insert was slightly thickened accordingly. A bushing ring inside the cardan shaft was also removed. The Type 2 and Type 3 has the same geometric design, and the details of all types are shown in Supplementary Figure S1.

However, both the cardan shaft and tibial component were made of CoCrMo. Although our previous study showed that the flexion shafts and cardan shaft are not the main bearing components (Guo and Guo, 2022), it is doubtful whether retaining a metal-on-metal interface after removing the PE gasket is safe and appropriate. This interface may not withstand repeated motion and may increase the risk of wear, especially considering the cytotoxicity of CoCrMo wear debris.

CF60-PEEK reinforced by continuous carbon fiber has shown a comparable elastic modulus (150 GPa) to CoCrMo (218 GPa), with reduced wear rates and cytotoxicity of wear debris. Therefore, to reduce the potential for wear while maintaining high stiffness, CoCrMo was replaced by CF60-PEEK for the cardan shaft, while the tibial component remained made of CoCrMo. Consequently, Type 2 (which contains a CoCrMo cardan shaft) served only as a control group in this study. Apart from the structural issues, the weight of the CoCrMo components also drew our attention. The advantages of using CF30-PEEK in tumor-type prostheses were confirmed in our previous study (Guo and Guo, 2022), and we retained the design of CF30-PEEK femoral component and extension rod in Type 3 (Figure 1B).

### 2.2 Finite element model construction

The CT data was derived from the same patient as our previous study (Guo and Guo, 2022), with a height of 168 cm and a weight of 70 kg. The total femur and total tibia measured 430 mm and 330 mm in length, respectively. Next, the medical modeling software Mimics 20.0 (Materialise Inc., Belgium) was used to construct the original knee joint model. The osteotomy length for the resection of the distal femur tumor was set at 128 mm, and for the proximal tibia it was set at 10.5 mm. Both osteotomy directions were perpendicular to the axis of the medullary cavity.

Subsequently, the simulated osteotomy was performed, and a custom prosthesis model was designed using engineering design software CREO 7.0 (PTC, United States). To analyze the stress distribution of three different implants in two static postures (knee joint at 0° for standing and at 90° for squatting), a finite element (FE) environment was created. The Altair Inspire software (Altair Engineering Inc., United States) was used for the pre-processing of this environment. Static structural analysis was performed using the OptiStruct 2019 solver (Altair Engineering Inc., United States). All materials were simplified to be linear elastic and isotropic, and the properties of different materials are shown in Table 1.

**TABLE 1:** The characteristics of different materials.

Materials	Young’s modulus (MPa)	Poisson’s ratio	Tensile strength (MPa)	Compressive strength (MPa)
Cortical bone Rezwan et al. (2006), Guo and Guo (2022)	14,000	0.30	50–151	130–220
CoCrMo Sevimay et al. (2005), Herranz et al. (2020)	218,000	0.33	409–431	632–682
Ti6Al4V Arrazola et al. (2009), Kaleli et al. (2018), Ishfaq et al. (2021)	110,000	0.35	960–1,100	870–1,010
UHMWPE Manoj Kumar et al. (2015), Malito et al. (2018)	1,016	0.30	10.2–26.3	9.8–15.7
CF30-PEEK Sarot et al. (2010), Schwitalla and Müller (2013)	18,000	0.39	175–214	239–246
CF60-PEEK Schwitalla et al. (2015), Liao et al. (2020)	150,000	0.35	2000	800

### 2.3 Loading and boundary conditions

In the loading condition, a downward load of 700 N was applied vertically to the femoral head to simulate the body’s gravity in one-legged standing. A load of 2,800 N was applied to simulate the highest loaded condition during one-legged squatting (Smith et al., 2008; Kutzner et al., 2010; Bergmann et al., 2014). In both postures, the distal end of tibia was fully constrained in all directions (Supplementary Figures S2A, B).

As shown in Supplementary Figures S2C, D, the contact conditions were varied among standard, boned and no contact. Briefly, the interfaces between the intramedullary stems and bones were bonded due to the bone cement design (indicated in blue). The interfaces between the femoral intramedullary stem, extension rod and the femoral component were also bonded because of the taper connection. For the remaining interfaces, standard contact conditions were applied (indicated in green). Additionally, some seemingly narrow gaps did not actually result in contact based on the geometric design (indicated in gray).

### 2.4 Model verification and data processing

Initially, the 10 Nodes tetrahedral (TET10) elements were utilized for calculations. However, during the gradual mesh refinement, increasing errors emerged and prevented the completion of the calculations. This issue could be attributed to the complexity of the model, which includes multiple components with intricate structures. Additionally, the software may automatically adjust meshes to accommodate small curvature corners, potentially distorting the meshes, which could significantly impact the stress levels and yield unreliable results (Saadlaoui et al., 2017).

For computational efficiency, the 4 Nodes tetrahedral (TET4) elements was subsequently employed for the calculation. In this study, the von Mises stress was selected as the primary parameter for evaluating biomechanical performance of the prosthesis. A convergence test was conducted on both Type 1 and Type 3 models by increasing the mesh densities (Supplementary Figures S3A, B). It was observed that convergence curve was not smooth. This could be attributed to the fact that many parameters of the model could affect mesh convergence behavior, and convergence tests may not always yield satisfactory solutions (Schmidt et al., 2009).

Another reason for the lack of smoothness in the convergence curve may be that the models retained substantial details, resulting in different convergence rates at different locations (Schmidt et al., 2009). For instance, considering the femoral component, which is of particular interest in this study, as the mesh density increased, the smooth surfaces of the medial and lateral condyles converged at much lower mesh densities. In contrast, the intercondylar fossa with curvature features exhibited slower convergence rates (Supplementary Figures S3C, D). Theoretically, the best way to determine whether smaller element sizes would result in smooth curves without abrupt changes would be to continuously increase the mesh densities, although this approach is impractical (Bright and Rayfield, 2011).

The different rate was calculated as the percentage difference in the maximum von Mises stress between the current mesh density and the previous density (Supplementary Tables S1, S2). Regarding the different rate, there appears to be no consensus in the literature, with values of <10% or <5% both being quoted (Schmidt et al., 2009; Bright and Rayfield, 2011; Lai et al., 2015; Tseng et al., 2015). In this study, the convergence was considered to have started at where a <10% different rate was observed between successive meshes. Consequently, for most locations, the Type 1 model can be deemed to have converged at 341,411 elements and the Type 3 model at 333,723 elements, with an average mesh size of 2.0 mm (Table 2).

**TABLE 2:** The number of nodes and elements for each model.

	Model	Nodes	Elements
Standing	Type-1	83,163	341,411
	Type-2	80,812	333,723
	Type-3	80,812	333,723
Squatting	Type-1	83,180	339,746
	Type-2	81,739	337,572
	Type-3	81,739	337,572

All the statistical analyses in this study were performed with the SPSS 22.0 software. For comparison of three groups, one-way ANOVA was performed. The differences were considered significant at *p* < 0.05.

## 3 Results

### 3.1 Development of micro-motion tumor prosthesis

**FIGURE 2:**
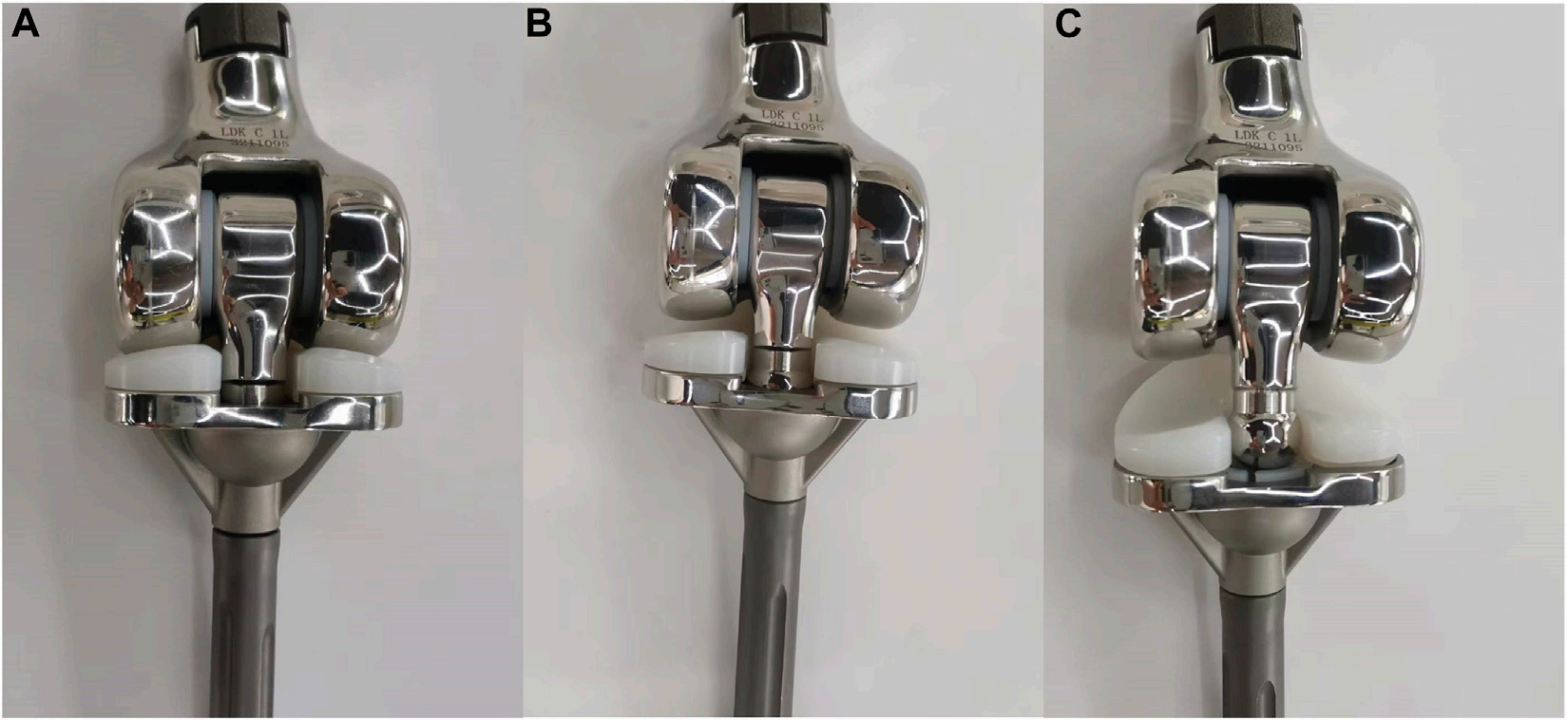
Illustration of **(A)** a normal Type 1 prosthesis, **(B)** the cardan shaft being pulled out from the cardan gasket, and **(C)** the cardan shaft fully dislocated and stuck into the tibial insert.

The initial design (referred to as Type 1) was first demonstrated in our previous study (Guo and Guo, 2022). For individuals in Asia and the Middle East, certain daily activities such as toileting, kneeling, and cross-legged sitting often require full flexion squatting (Hemmerich et al., 2006). However, during the subsequent static loading test of Type 1, which simulated this specific movement, unsatisfactory results were observed. It was discovered that there was a potential issue where the cardan shaft could be pulled out from the cardan gasket, leading to subluxation, or even getting stuck in the gap of the tibial insert, resulting in complete dislocation (Figure 2). This occurred when the prosthesis underwent prolonged bending at a high flexion angle with load-bearing. If this situation were to occur in a clinical utilization, it would require a revision surgery to restore the functionality of the knee joint. To address this issue, the cardan shaft was redesigned as described in the Methods section. Subsequently, the stress distribution of all three prostheses was analyzed.

### 3.2 Overall stress distribution and displacement of three models

**FIGURE 3:**
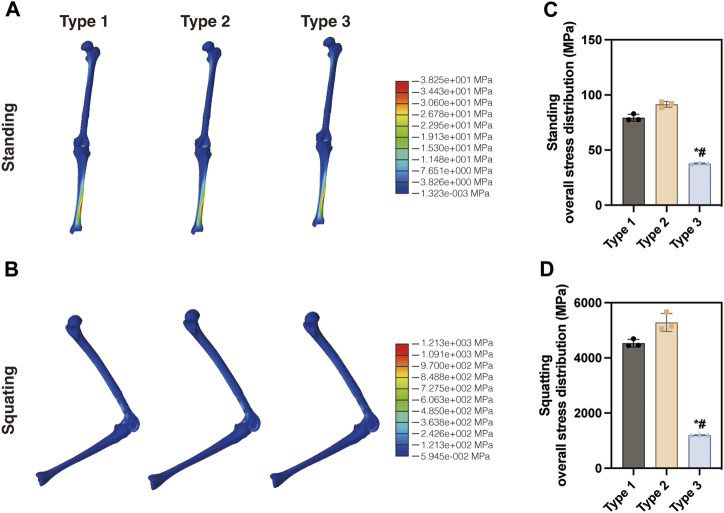
Distribution of the von Mises stress in the three entire models in the **(A)** standing position and **(B)** squatting position. Statistical analysis of the maximum von Mises stress in the **(C)** standing and **(D)** squatting position (*p < 0.05 compared with Type 1, #*p* < 0.05 compared with Type 2, same in the following figures).

**FIGURE 4:**
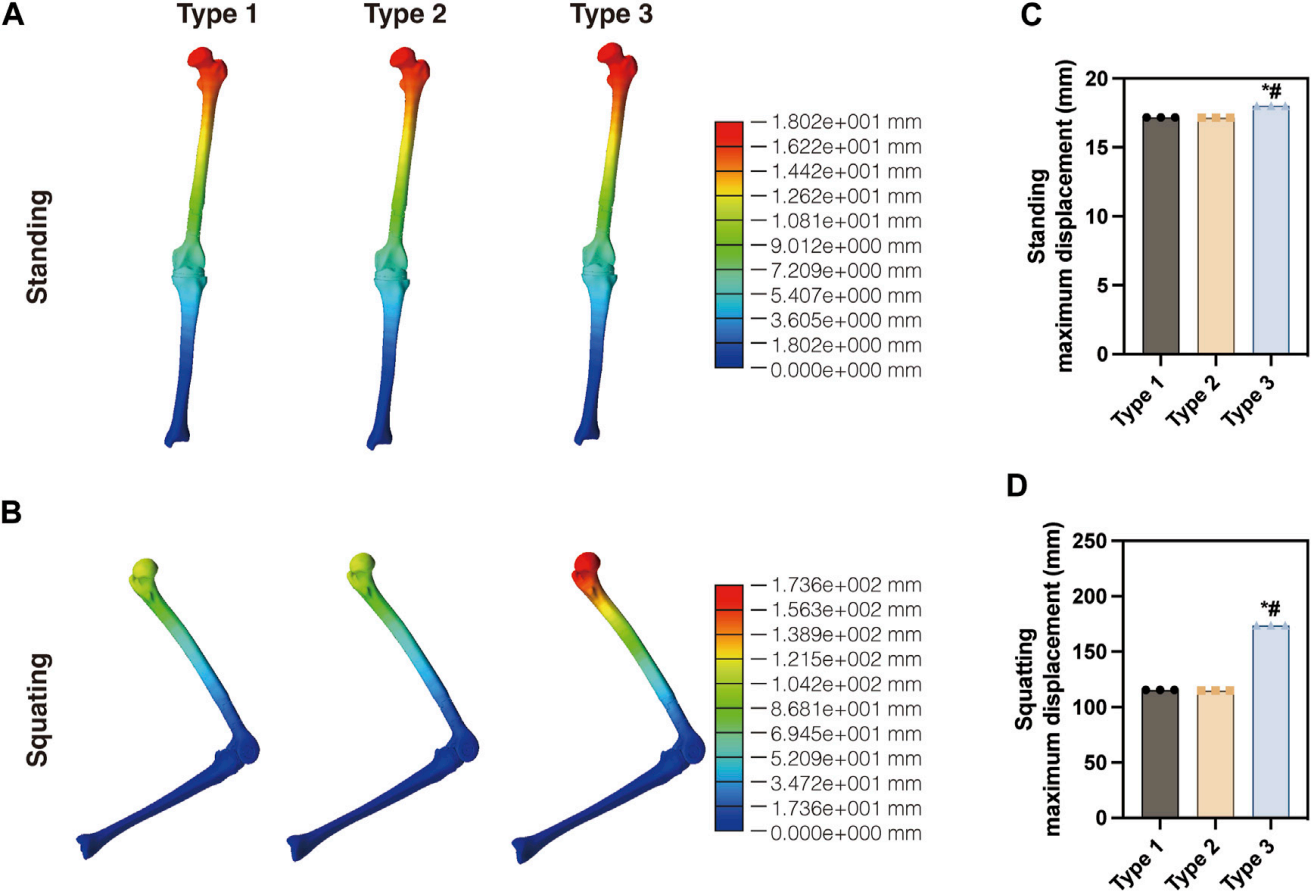
Displacement of the three models in the **(A)** standing and **(B)** squatting position. Statistical analysis of the maximum displacement in the **(C)** standing and **(D)** squatting position.

The von Mises stress distribution of three models, including the proximal femur and tibia, was analyzed in this study. Figure 3A illustrates the overall stress distribution of the entire model in the standing posture. The maximum stress value was observed in Type 2 (93.63 MPa), which was higher than Type 1 (82.95 MPa) and Type 3 (38.25 MPa). As shown in Figure 3B, when squatting, Type 2 showed the highest stress value (5,662 MPa), significantly exceeding that of Type 1 (4,694 MPa) and Type 3 (1,213 MPa). For a more detailed examination, three random points around the maximum stress point were collected for statistical analysis. As shown in Figures 3C, D, the maximum stress observed in the Type 3 model was the lowest in both standing and squatting position. Compared with Type 1, Type 3 showed a distinct reduction of 53.9% in maximum von Mises stress during standing and 74.2% reduction during squatting. The maximum displacement of the entire model was shown in Figure 4. The maximum displacement was observed in the proximal femur in both positions. When standing, the maximum displacement in Type 3 (18.02 mm) was slightly higher than that of Type 1 (17.48 mm) and Type 2 (17.53 mm). In the squatting position, the maximum displacement remained highest in Type 3 (173.6 mm), significantly surpassing that of Type 1 (115.5 mm) and Type 2 (114.8 mm).

### 3.3 Mechanical analysis of extension rod, femoral component and tibial insert

**FIGURE 5:**
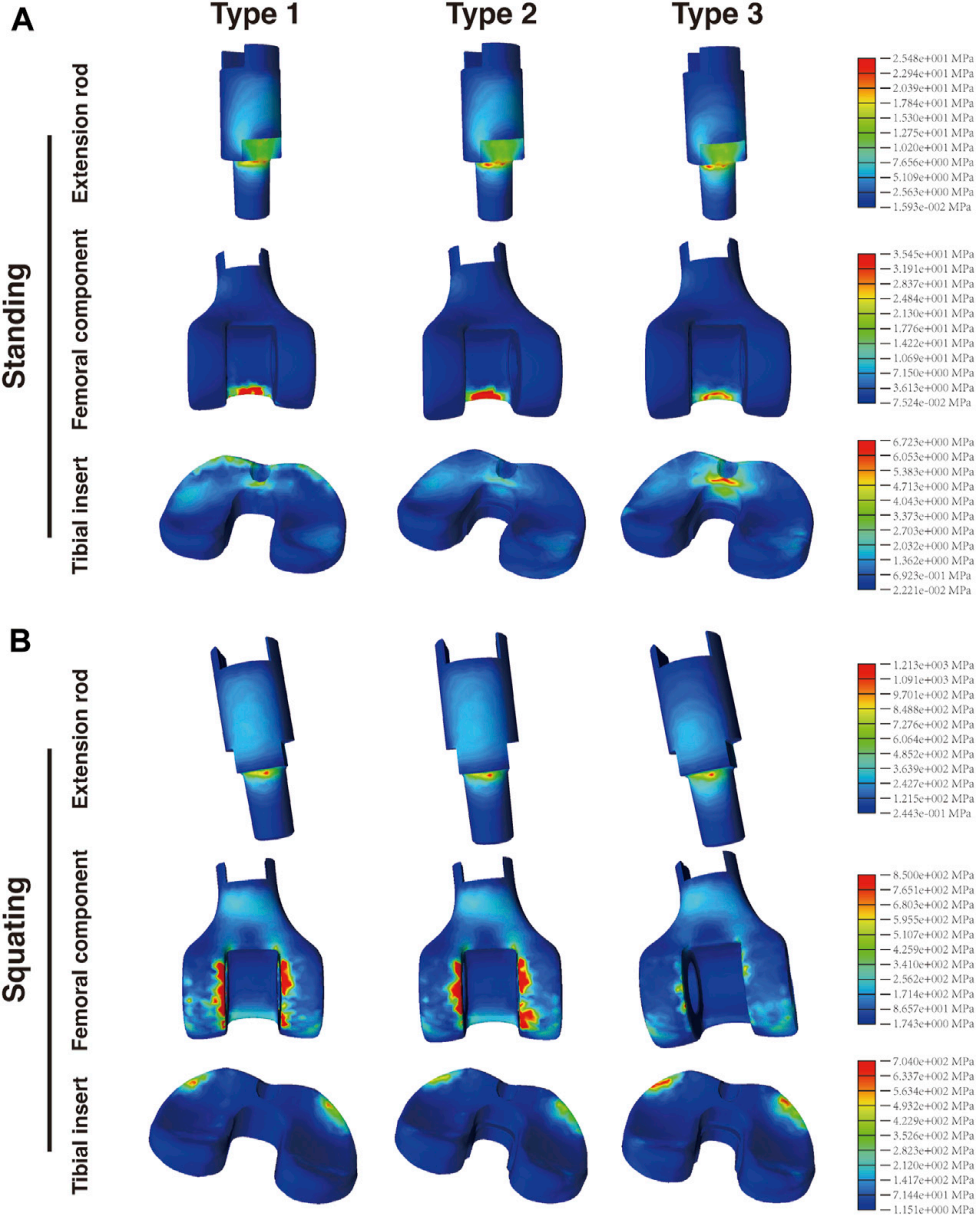
Distribution of the von Mises stress in the extension rod, femoral component and tibial insert in the **(A)** standing and **(B)** squatting position.

The extension rod and femoral component of three prostheses were made of materials with different mechanical properties, which could potentially affect the stress distribution. However, as shown in Figure 5, in the standing condition, the maximum stress values observed on the extension rod were nearly identical for Type 1 (22.15 MPa), Type 2 (26.83 MPa), and Type 3 (25.48 MPa). This trend remained similar in the squatting position, with values of 1,308, 1,283, and 1,213 MPa, respectively.

There were significant differences in the stress distribution on the femoral component. In the standing condition (Figure 5A), the maximum stress on the CF30-PEEK femoral component in Type 3 (35.45 MPa) was significantly lower than that of the CoCrMo component in Type 1 (82.95 MPa) and Type 2 (93.63 MPa). Moreover, in all three types, the highest stress on the femoral component appeared at the contact area with the cardan shaft, located within the region corresponding to intercondylar fossa. In the squatting condition, the maximum stress on the femoral component of Type 3 (850 MPa) was substantially lower than that of Type 1 (4,694 MPa) and Type 2 (5,662 MPa). It is noteworthy that the maximum stress in all three types of prostheses was observed at the interface between the femoral component and tibial insert (Figure 5B). Compared with Type 1, the maximum stress on the femoral component of Type 3 decreased by 57.3% and 81.9% under the standing and squatting conditions, respectively.

Regarding the tibial insert, stress concentration was observed on the anterior regions of the insert in all three types. The maximum stress observed in Type 3 (6.723 and 704 MPa) was higher than that of Type 1 (4.095 and 625.1 MPa) and Type 2 (3.853 and 519.3 MPa) in both postures (Figures 5A, B). Further analysis of the medial and lateral sides of the insert (corresponding to the medial and lateral menisci) revealed that the maximum von Mises stress on both sides of the tibial insert in Type 3 was also higher than those in the other two types (Supplementary Figure S4).

### 3.4 Stress analysis of connecting components

**FIGURE 6:**
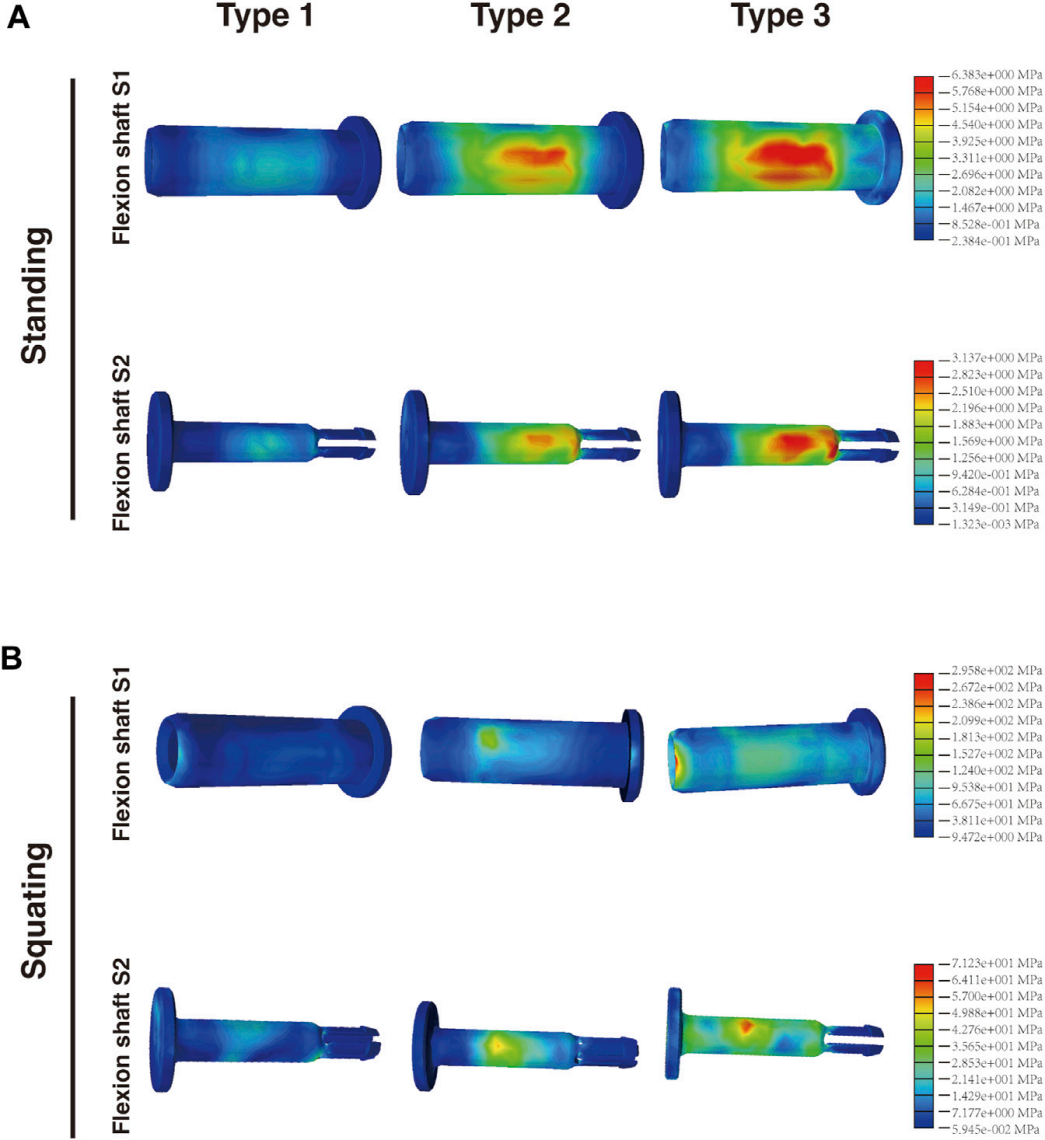
Distribution of the von Mises stress on flexion shaft S1 and S2 in the **(A)** standing and **(B)** squatting position.

**FIGURE 7:**
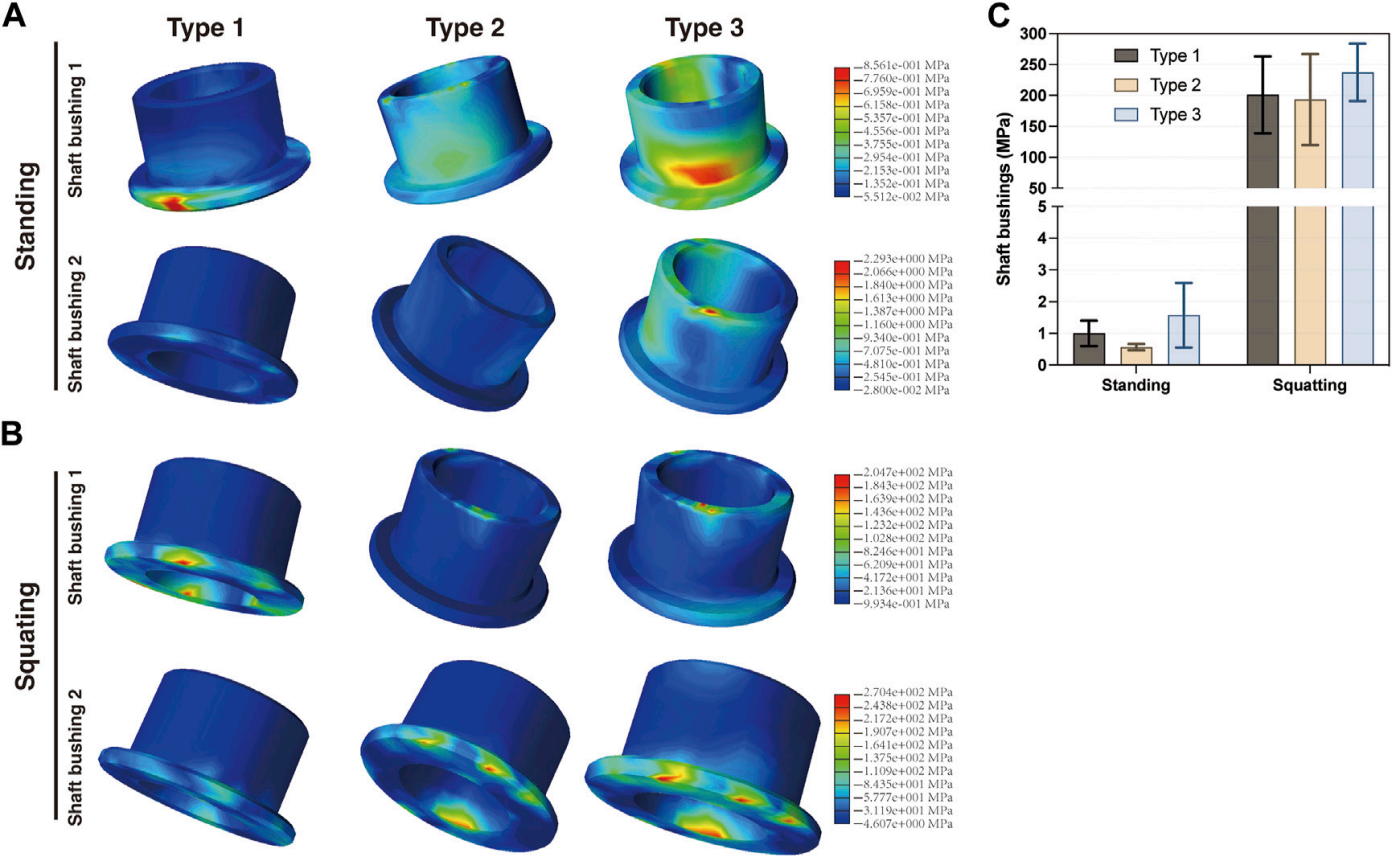
Distribution of the von Mises stress on shaft bushings in the **(A)** standing and **(B)** squatting position, and **(C)** statistical analysis of the maximum stress in three types.

The connecting components consist of the flexion shaft S1 and flexion shaft S2 (Figure 1). S2 is inserted into S1, with two shaft bushings positioned between S1 and the femoral component. Together, these connecting components articulate the cardan shaft with the femoral component. When standing, the maximum stress on both the flexion shaft S1 (6.383 MPa) and S2 (3.137 MPa) of Type 3 was slightly higher than that of Type 1 (2.158 and 1.148 MPa, respectively) and Type 2 (5.842 and 2.545 MPa, respectively) (Figure 6A). Similarly, when squatting, Type 3 exhibited the highest stress on S1 (295.8 MPa) and S2 (71.23 MPa) among the three types, as compared with Type 1 (105.7 and 36.51 MPa, respectively) and Type 2 (169.8 and 54.06 MPa, respectively) (Figure 6B). In the standing condition, most stress located on the central area of shaft S1 and S2 in all the three types. However, when squatting, most stress was concentrated on the central area of S2 but a greater amount was found on the edge of S1. The maximum stress on all shaft bushings showed no significant differences among them, although Type 3 presented slightly higher stress values regardless of position (Figure 7).

### 3.5 Stress analysis of micro-motion components

The cardan shaft is a key distinguishing feature that sets this prosthesis apart from other distal femoral tumor-type prostheses. It serves the dual purpose of connecting the femoral and tibial components and enabling micro-motions in multiple directions, thus preserving the flexibility of the knee joint.

**FIGURE 8:**
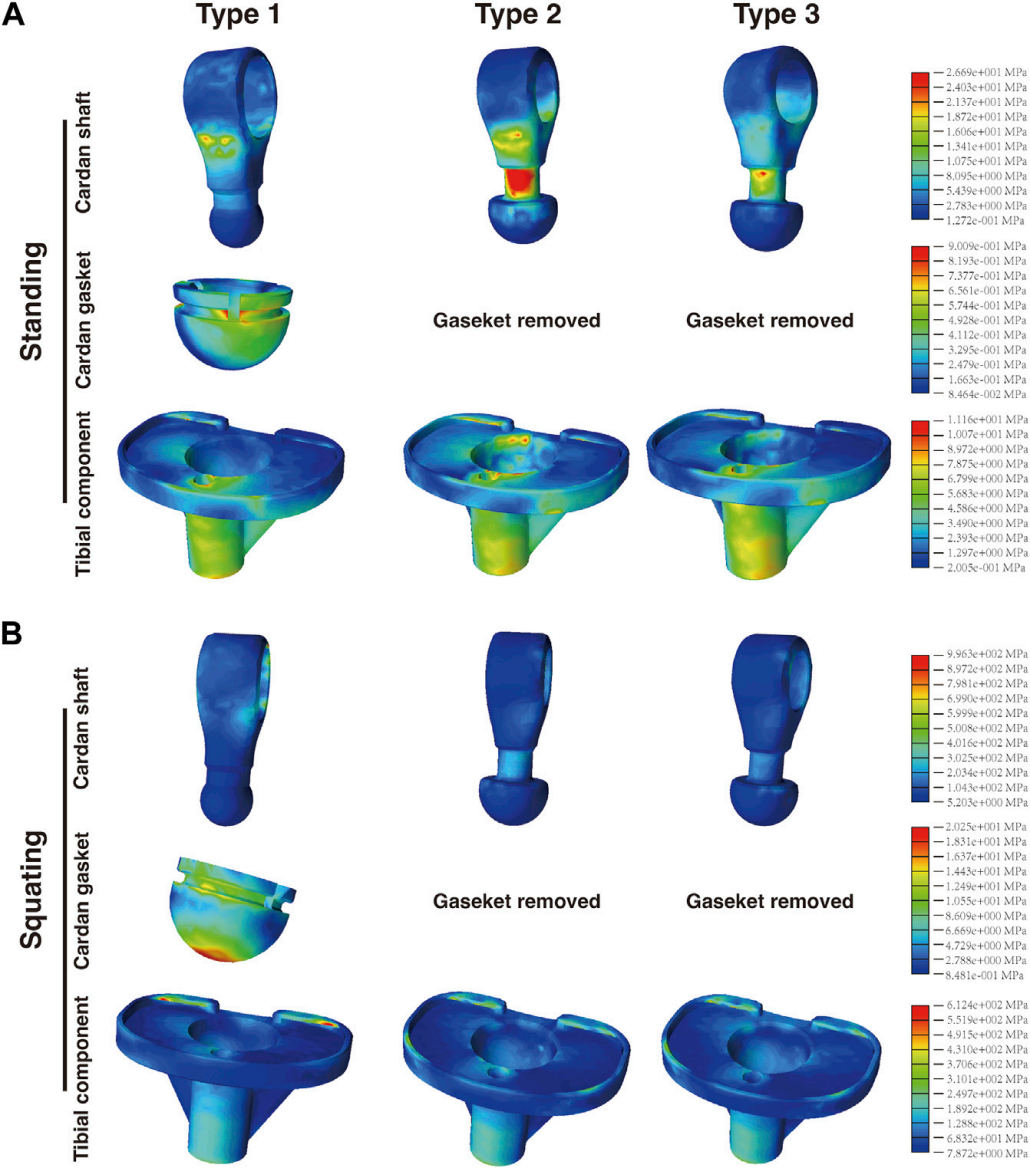
Distribution of the von Mises stress on the cardan shaft, cardan gasket, and tibial component in the **(A)** standing and **(B)** squatting position.

Figure 8 demonstrates that in Type 2 and Type 3, the cardan gasket was removed due to the enlarged distal end of the cardan shaft. Following design and material adjustments, it was observed that when standing, the maximum stress in the cardan shaft of Type 3 (26.69 MPa) was slightly higher than that of Type 1 (22.29 MPa) but lower than Type 2 (37.34 MPa). In Type 1, the majority of the stress was located at the contact area with femoral component, whereas in Type 2 and Type 3, more stress appeared on the neck of the cardan shaft. In the squatting position, the stress distribution differed from the standing posture. The maximum stress in Type 3 (996.3 MPa) was significantly lower than that in Type 1 (1862 MPa) but similar to Type 2 (970.3 MPa). The majority of high-stress was located at the flange region of upper cardan shaft, in contact with the shaft bushings, with no significant stress concentration in the neck or bottom areas. Compared with Type 1, the maximum stress on the cardan shaft of Type 3 increased by 19.7% when standing, but decreased by 46.5% when squatting.

Regarding the cardan gasket in Type 1, most of the maximum stress was observed on the edges below the top of the gasket. When squatting, stress concentration appeared at the central bottom of the gasket, which was not obvious when standing. Furthermore, in Type 1, the gasket top was in contact with the tibial insert, presenting an interface stress of approximately 0.16–0.53 MPa in the standing position and 2.06–8.52 MPa in the squatting position. It is worth noting that in Type 2 and Type 3, the upper surface of the hemisphere structure was not in contact with the bottom of the tibial insert (Supplementary Figure S1), thus showing a normal stress gradient.

Analysis of the tibial component revealed that when standing, the maximum stress in Type 3 (11.16 MPa) was lower than that of Type 1 (20.62 MPa) and Type 2 (15.78 MPa). When squatting, Type 1 showed the highest stress value of 937.8 MPa, followed by Type 3 (612.4 MPa) and Type 2 (532.1 MPa). The distribution of von Mises stress on the tibial component was similar among the three types in the standing or squatting position, respectively.

### 3.6 Stress analysis of femur, tibia, and intramedullary stems

**FIGURE 9:**
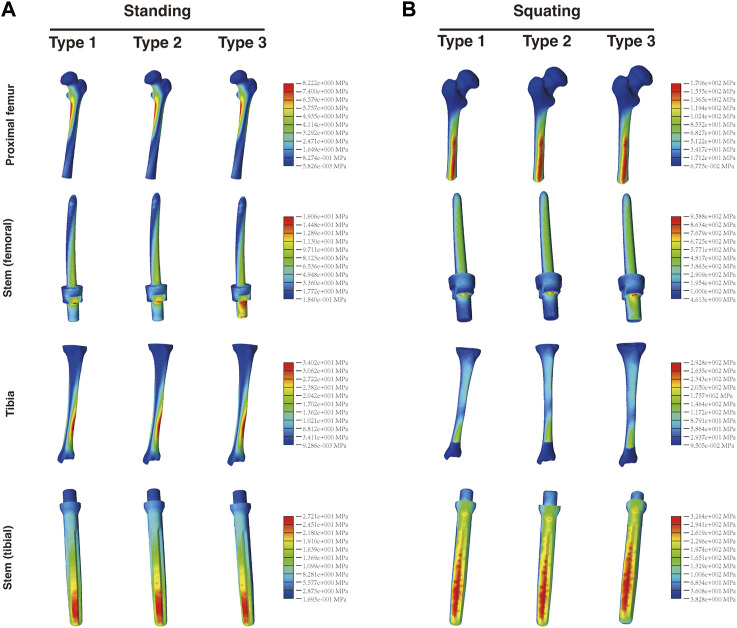
Distribution of the von Mises stress on the proximal femur, tibia, and respective intramedullary stems in the **(A)** standing and **(B)** squatting position.

**FIGURE 10:**
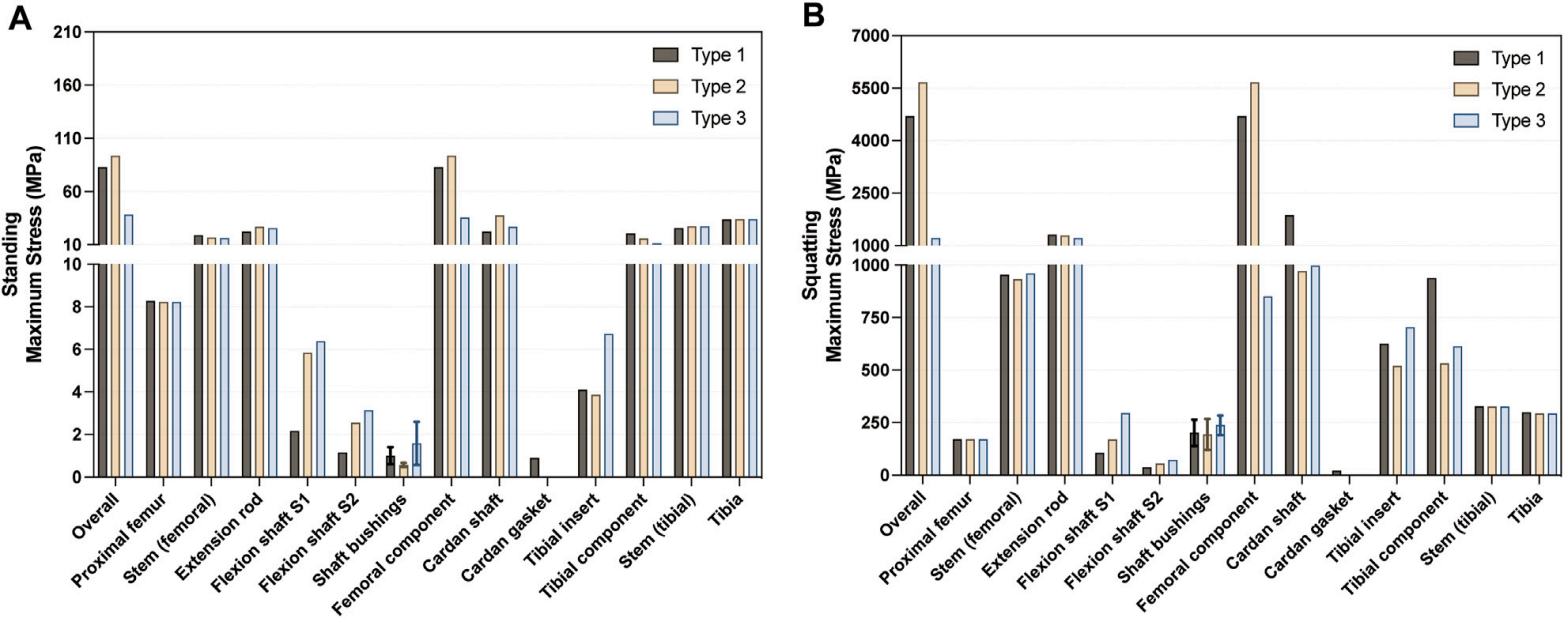
Summary of the maximum von Mises stress in each component in the **(A)** standing and **(B)** squatting position.

Figure 9 demonstrates that in the standing condition, the maximum stress on the proximal femur was nearly identical between Type 1 (8.262 MPa) and Type 2 and Type 3 (both 8.222 MPa). Additionally, the maximum stress observed on the tibia was similar among Type 1 (33.61 MPa) and the other two types (both 34.02 MPa). Furthermore, there were only minor differences in the stress distribution among the femoral or tibial intramedullary stems, respectively. A similar trend was observed in the squatting condition. These findings suggest that the adjustments in design and material had minimal impact on the stress distribution of the femur, tibia, and their intramedullary stems. Figure 10 presents the maximum stress values of each component in all three types.

## 4 Discussion

The wide resection of bone tumors around the knee requires reconstruction with appropriate tumor-type prostheses. In our previous study (Guo and Guo, 2022), we proposed the initial design of a micro-motion tumor-type knee prosthesis. This prosthesis incorporates a crucial cardan shaft connection, which enables micro-motion between the femur and tibia, preserving the flexibility of the knee joint after implant surgery. However, during subsequent tests simulating prolonged squatting with weight-bearing, the prosthesis exhibited a potential for dislocation, leading to malfunctions of the prosthesis. Consequently, a redesign of the prosthesis became necessary. The primary cause of dislocation was the cardan shaft being pulled out from the shaft gasket and the tibial component. Therefore, the redesign primarily focused on addressing this issue through modifications to the cardan shaft, as described above.

CFR-PEEK has been successfully used in many orthopedic reconstructions. Although CF30-PEEK has not been extensively used for femoral components or load-bearing parts of joint prostheses (Koh et al., 2019; Vertesich et al., 2022), it has demonstrated good load-bearing capacity in other clinical applications and has shown stable long-term performance (Nakahara et al., 2013; Rotini et al., 2015; Schliemann et al., 2015; Boriani et al., 2018; Laux et al., 2018; Ziegler et al., 2019). Additionally, our previous study found favorable stress distribution in the femoral component made of CF30-PEEK (Guo and Guo, 2022). These findings suggest that CF30-PEEK can potentially replace CoCrMo for the femoral component, reducing the weight of the tumor-type prosthesis without compromising its strength. Therefore, in this study, we chose to retain CF30-PEEK as the material for the femoral component in Type 3.

By adjusting the arrangement and weight fraction of carbon fibers embedded in CFR-PEEK composites, CF60-PEEK exhibits an elastic modulus ranging from 50 to 150 GPa (Schwitalla et al., 2015; Xu et al., 2019; Liao et al., 2020; Souza et al., 2021). This allows for achieving higher rigidity and torsion resistance similar to metals, while maintaining a lighter weight (Verma et al., 2021). Clinical use of long carbon fiber-reinforced PEEK composites, such as the Carbofix Piccolo system, have demonstrated good load-bearing capabilities and bending resistance (Hak et al., 2014). Tests conducted by Steinberg et al. showed that tibial nails and plates made of 60wt% CFR-PEEK exhibit similar mechanical characteristics to commercially available metal implants, with lower wear performance (Steinberg et al., 2013). CF60-PEEK also demonstrates excellent wear resistance (Koh et al., 2019). The wear rate of CFR-PEEK is lower than that of metal-on-UHMWPE and metal-on-metal systems (Howling et al., 2003; Scholes and Unsworth, 2009a; Scholes and Unsworth, 2009b). Considering these characteristics, CF60-PEEK was selected as the material for the cardan shaft in this study to ensure component rigidity, stability, and high wear resistance. Its impact on stress distribution was subsequently analyzed.

Finite element analysis (FEA) is a highly effective and powerful tool for evaluating multiple variables in orthopedic implants, aiding in design optimization and predicting stress distribution (Pfeiffer, 2016). In this study, three different finite element models were developed, incorporating Young’s modulus and Poisson’s ratio to complete the models. To independently assess the effect of material variations on stress distribution, the Type 2 group, which had the same geometric structure as Type 3 but retained the CoCrMo composition as Type 1, served as control group as well. However, there is currently a lack of consensus on the loading or contact stress on the knee joint during movement, particularly after reconstruction with a tumor-type knee prosthesis. Research on the knee joint suggests that the peak axial force during level walking can range from 2.2 to 2.5 times body weight (BW) (Bergmann et al., 2014). In fast walking, the medial knee contact force can increase by 30%–70% compared to the standing position, and the loading is greatly influenced by muscle force (Ogaya et al., 2015; Trepczynski et al., 2018). Stair descent can generate forces up to 346% BW, while stair ascent can result in forces up to 316% BW (Kutzner et al., 2010). These values vary widely among individuals, and the estimation of contact force may have an error of 10% BW (Jung et al., 2017).

Furthermore, the load on the knee joint becomes more complex in the deep squat position (beyond 90 degrees of flexion). On one hand, the actual tibiofemoral contact forces depend on the net moments of the hamstrings or the quadriceps force in the sagittal plane (Smith et al., 2008; Kutzner et al., 2010; Bergmann et al., 2014). On the other hand, even at the same knee flexion angle, loading conditions on the knee joint can vary during activities such as stair ascent, rising from a chair, and deep squatting. For example, during one-legged flexion in stair ascending, the knee joint experiences forces equivalent to 316% BW, while during two-legged bending in deep squatting, the forces range from 240% to 253% BW (Taylor et al., 1998; Smith et al., 2008; Kutzner et al., 2010; Bergmann et al., 2014). Moreover, due to the various combinations of anatomical structures and complex movements, determining the normal loading conditions within the knee joint is challenging (Taylor et al., 2004).

Therefore, for computational efficiency, in our study, we applied a load of 700 N to simulate the load during standing, and 2800 N to simulate the extreme load during knee bending in the gait cycle. These loads are slightly higher than those used in previous finite element studies, but align more closely with the peak loading during one-legged postures (Bergmann et al., 2014), as we aimed to encompass a wider range of the complexity and variability of knee joint loading. However, considering that patients may use assistive devices such as braces or crutches during postoperative recovery, the load settings in this study may be higher than the actual tibiofemoral contact stress under most circumstances.

Research has shown that for primary distal femoral endoprostheses, decreased survivorship is primarily attributed to soft tissue failure, aseptic loosening, and structural complications (Pala et al., 2013; Bus et al., 2017; Haijie et al., 2018; Ogura et al., 2021). In the case of revision prostheses, periprosthetic fracture and aseptic loosening are the primary causes of implant failure (Pala et al., 2013; Geiger et al., 2023). In this study, the overall von Mises stress of the newly designed prosthesis was significantly lower than that of the other two groups in both the standing and squatting position. Stress analysis of the entire model showed that, after updating the design and material, the overall stress on the Type 3 prosthesis remained consistent and evenly distributed. Additionally, there was no significant difference in the stress distribution observed at the bone-prosthesis interface between Type 3 and Type 1 in both standing and squatting positions. The von Mises stress between the bones and intramedullary stems was similar, indicating no significant stress shielding effects. This suggests that the stress in the Type 3 model is effectively transmitted from the prosthesis to the cortical bone, preventing instability between the prosthesis and bone and avoiding subsequent complications.

The displacement analysis of the entire model revealed that in the standing position, Type 3 showed similar results to the other two groups. However, during the higher load-bearing squatting position, the displacement in Type 3 significantly increased. This can be attributed to the notable difference in stiffness between the CF30-PEEK components and the CoCrMo components. The extension rod and femoral component that made of CF30-PEEK exhibit lower stiffness but higher elastic deformability, which aligns with the findings of our previous study (Guo and Guo, 2022).

Upon further analysis of the load distribution on the components, it was observed that after the design modifications, there were no significant differences in the stress distribution of the intramedullary stems of the femur and tibia, extension rod, and bushings for flexion shafts. However, notable changes were observed in the stress distribution of the components that had been replaced with new materials and the components in contact with them. When comparing the stress distribution of the femoral components, it was found that in the standing position, the region in contact with the cardan shaft (referred to as the intercondylar fossa) showed the highest stress. In the squatting position, the maximum stress shifted to the region in contact with the tibial insert. The femoral component made of CF30-PEEK exhibited significantly lower maximum stress in both standing and squatting positions, compared with the CoCrMo groups. A long-term follow-up research showed that after tumor resection and reconstruction in the knee joint, the prosthesis failure was primarily caused by mechanical facts (Henderson et al., 2011). These failures are likely linked to the weight of the prosthesis. The findings in this study highlight the positive impact of using CF30-PEEK in the femoral component, as it not only effectively reduces the weight burden but also mitigates the risks of fatigue due to stress concentration.

Analysis of the tibial insert revealed complex results. The Type 3 group showed higher maximum stress on the UHMWPE insert compared to the other two groups, in both standing and squatting postures. Further analysis revealed that both the medial and lateral regions of the tibial insert experienced elevated stress in the Type 3 group. In this study, the elastic modulus of CF30-PEEK was set at 18 GPa, significantly lower than that of CoCrMo but closer to UHMWPE. This relative modulus compatibility helps with load transfer and promotes even stress distribution (Heary et al., 2016). It was further confirmed in this study, where the von Mises stress on the CF30-PEEK femoral component was closer to that of the UHMWPE insert, compared with the CoCrMo-UHMWPE pairs. These findings suggest that the load force could be effectively transmitted from the femoral component to the tibial insert. However, it is important to note that all three groups exhibited some stress concentration at the ridge of the tibial insert. Further research is necessary to assess the potential risks of wear and fatigue associated with tibial inserts.

The flexion shaft S1 and S2 play a crucial role in articulating the femoral components and the cardan shaft. The stress concentration regions on the flexion shaft may be influenced by the transmission of axial forces from the femoral component. Regarding the cardan shaft, when comparing Type 3 with Type 2 and Type 1, the utilization of CF60-PEEK as the material along with the design updates, resulted in a similar stress distribution when standing, but a substantial reduction in stress when squatting. This reduction could hold significant importance for tumor-type prostheses, as many of tumor-type prostheses still carry a risk of breakage (Yoshida et al., 2012). Studies have reported that approximately 2%–6% of tumor patients reconstructed with rotating hinge prostheses experienced fractures at the tibial yoke, where the rotating component was inserted into the tibial tray (Myers et al., 2007). In contrast to rotating hinge implants, in this prosthesis, the bottom of the cardan shaft remains unfixed, and stress is transmitted through the femoral component to the tibial insert and tibial component. We speculate that the substantial reduction in stress on the cardan shaft could help mitigate the risk of fractures resulting from increased activity and repetitive torsion.

As for the tibial component, Type 3 exhibited slightly higher stress, although the stress difference among the three groups was minimal. The region in contact with the tibial insert experienced the highest stress, and no stress concentration was observed at the contact surface between the cardan shaft and the groove of the tibial component. The low contact pressure contributes to reducing the risk of wear at the interface between the cardan shaft and the tibial component (Grupp et al., 2010; Koh et al., 2019).

In the squatting position, certain components showed stress levels that exceeded their theoretical yield strength. However, this does not necessarily indicate component failure. The results are influenced by various parameters such as mesh density and loading conditions. Utilizing loading conditions that are closer to physiological conditions could likely yield a more realistic stress distribution. We also acknowledge the following limitations in this study. Firstly, for computational efficiency, only Poisson’s ratio and elastic modulus were considered as parameters. Incorporating additional parameters for model construction (Viceconti et al., 2005) and using viscoelastic models for specific components may offer more comprehensive insights into stress distribution (Alotta et al., 2018). Secondly, this study exclusively assessed the von Mises stress on bones and the prosthesis under two static conditions. Future research should account for the gradual changes in a post-surgery patient’s gait. Incorporating dynamic motion models and analyzing the complete gait cycle would facilitate a more specific evaluation of stress distribution across a wider range of knee flexion degrees. Furthermore, a comprehensive biomechanical analysis should consider the influence of soft tissues and muscles, as they significantly impact joint loading. Additionally, combining the findings with *ex vivo* validations such as pressure-sensitive film tests would provide a more accurate assessment of the contact forces and stress distribution within the prosthesis (Khosravipour et al., 2018).

## 5 Conclusion

In conclusion, our study provides a novel investigation into a tumor-type prosthesis composed of CFR-PEEK, with a focus on assessing the von Mises stress distribution across its components. The results indicate that CF30-PEEK effectively transmits forces, leading to reduced stress concentration on the femoral component, while reduced weight and improved functionality. The incorporation of CF60-PEEK in the redesigned cardan shaft significantly reduces the von Mises stress while maintaining comparable stiffness. These advancements in force transmission and stress reduction are expected to enhance the stability and durability of the new tumor-type knee prosthesis. However, before introducing the prosthesis into routine clinical practice, further objective investigations on deformation and wear performance are necessary.

## Data Availability

The original contributions presented in the study are included in the article/Supplementary Material, further inquiries can be directed to the corresponding author.
