# Supplementary material for: Effect of carbon-fiber-reinforced polyetheretherketone on stress distribution in a redesigned tumor-type knee prosthesis: a finite element analysis
Source: Front Bioeng Biotechnol. 2023 Sep 26;11:1243936. doi: 10.3389/fbioe.2023.1243936 (PMC10562634; doi:10.3389/fbioe.2023.1243936)
Supplement: Supplementary file 1 [file Table1.docx]

Supplement Table 1. The maximum von Mises stress (MPa) and different rate (%) of Type 1 entire model at 700 N for different mesh densities.

| Mesh Density | Maximum von Mises stress (MPa) | Different Rate (%) |
| --- | --- | --- |
| 47009 | 63.93 | - |
| 58326 | 105 | 64% |
| 72579 | 74.83 | 29% |
| 98100 | 65.61 | 12% |
| 174264 | 52.56 | 20% |
| 192009 | 56.49 | 7% |
| 206142 | 81.15 | 44% |
| 276415 | 65.14 | 20% |
| 341411 | 82.95 | 27% |
| 496963 | 77.69 | 6% |
| 565925 | 75.22 | 3% |
| 904376 | 70.34 | 6% |
| 1239326 | 76.71 | 9% |
