# Supplementary material for: Effect of carbon-fiber-reinforced polyetheretherketone on stress distribution in a redesigned tumor-type knee prosthesis: a finite element analysis
Source: Front Bioeng Biotechnol. 2023 Sep 26;11:1243936. doi: 10.3389/fbioe.2023.1243936 (PMC10562634; doi:10.3389/fbioe.2023.1243936)
Supplement: Supplementary file 2 [file Table2.docx]

Supplement Table 2. The maximum von Mises stress (MPa) and different rate (%) of Type 3 entire model at 700 N for different mesh densities.

| Mesh Density | Maximum von Mises stress (MPa) | Different Rate (%) |
| --- | --- | --- |
| 44654 | 123.8 | - |
| 54818 | 134 | 8% |
| 67785 | 52.86 | 61% |
| 93946 | 39.7 | 25% |
| 134627 | 61.74 | 56% |
| 169211 | 32.01 | 48% |
| 201395 | 38.24 | 19% |
| 269000 | 33.39 | 13% |
| 333723 | 38.25 | 15% |
| 417284 | 37.01 | 3% |
| 646450 | 38.52 | 4% |
| 868185 | 39.36 | 2% |
| 1067964 | 36.51 | 7% |
