# Supplementary material for: Effect of carbon-fiber-reinforced polyetheretherketone on stress distribution in a redesigned tumor-type knee prosthesis: a finite element analysis
Source: Front Bioeng Biotechnol. 2023 Sep 26;11:1243936. doi: 10.3389/fbioe.2023.1243936 (PMC10562634; doi:10.3389/fbioe.2023.1243936)
Supplement: Supplementary file 3 [file DataSheet1.DOCX]

Supplementary Material

**
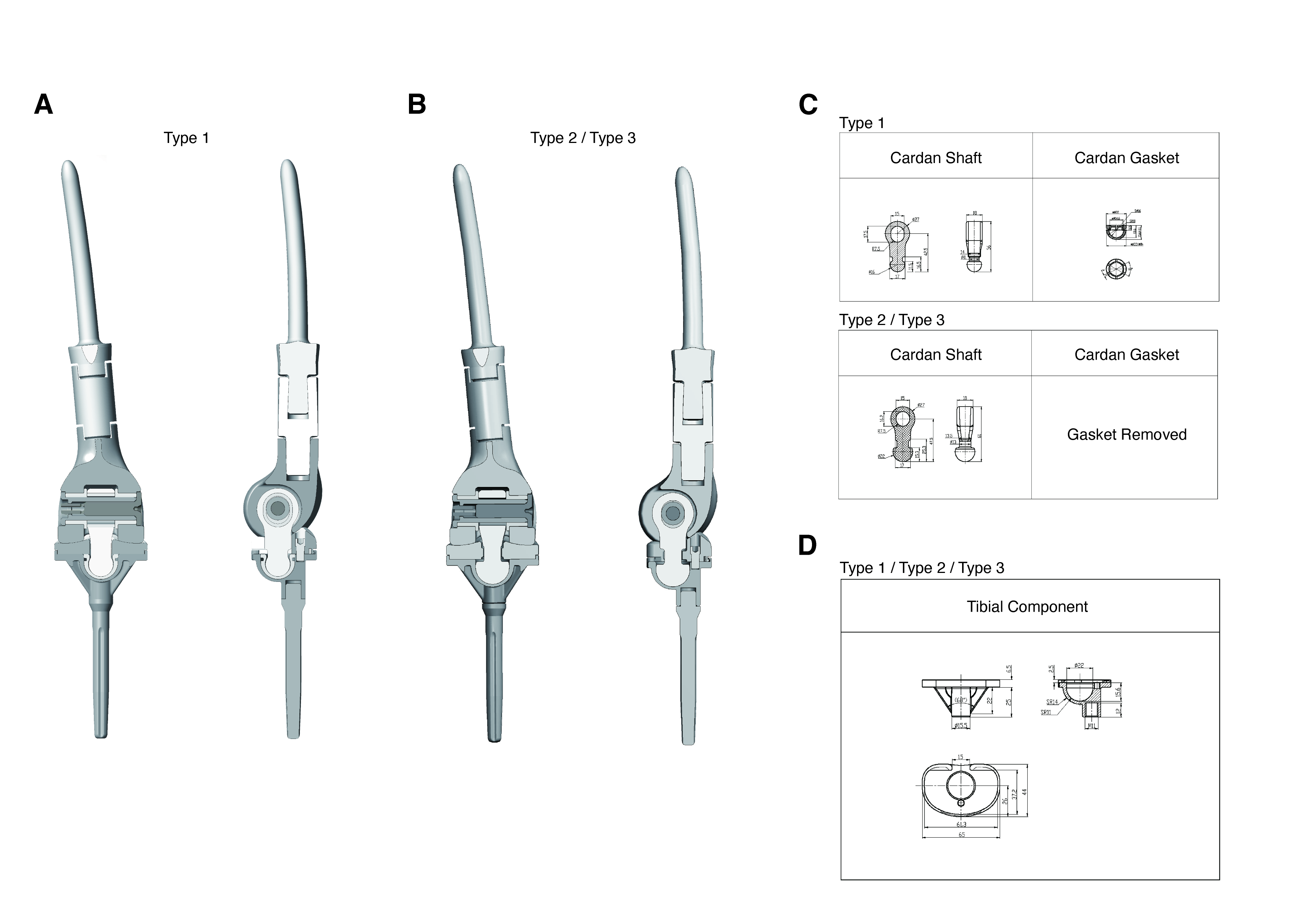
**

**Supplementary Figure 1.** Cross-sections of engineering drawings of the (A) original Type 1 prosthesis and (B) redesigned Type 2/ Type 3 micro-motion tumor-type knee prosthesis. Geometric details of (C) the cardan shaft of each type and (D) the tibial component.


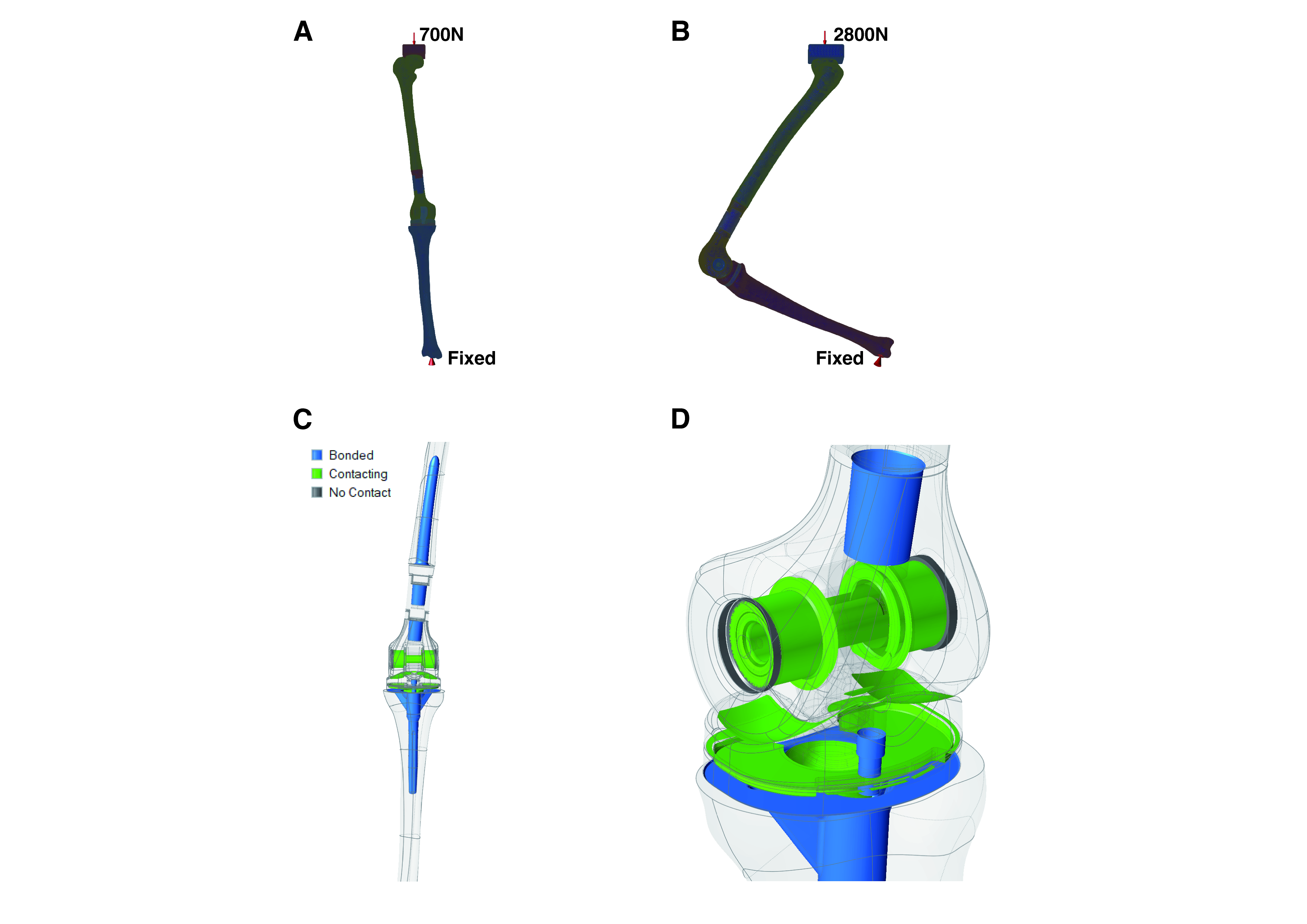


**Supplementary Figure 2.** Loading and boundary conditions of (A) standing and (B) squatting position. (C,D) Overview of the contact conditions, which are indicated with different colors: bonded (blue), standard contact (green), and no contact (gray).


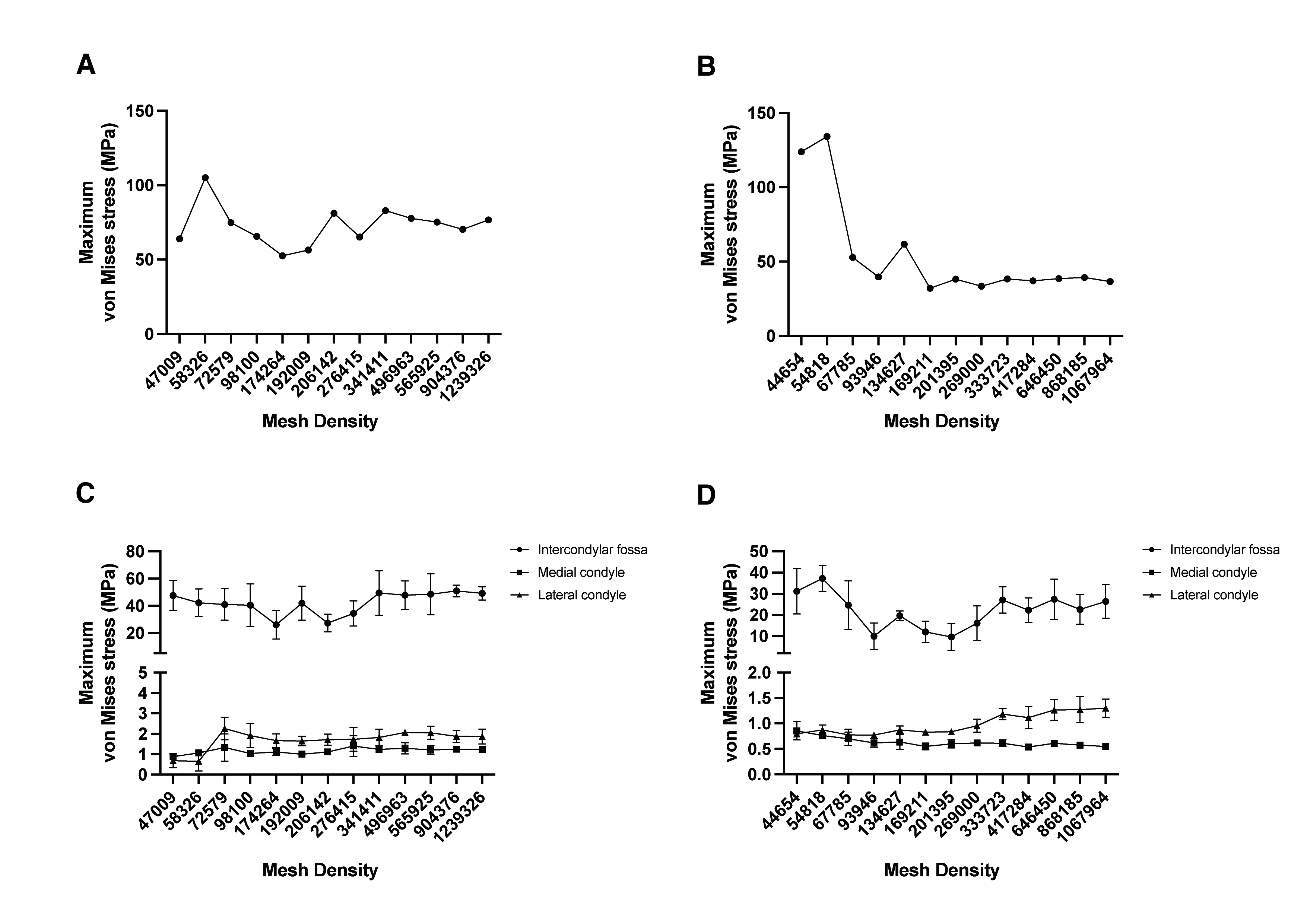


**Supplementary Figure 3.** Maximum von Mises stress of the (A) Type 1 and (B) Type 3 whole model in relation to the mesh density. Further analysis into the maximum von Mises stress of the intercondylar fossa, medial condyle and lateral condyle of the femoral component in (C) Type 1 and (D) Type 3 in relation to the mesh density.


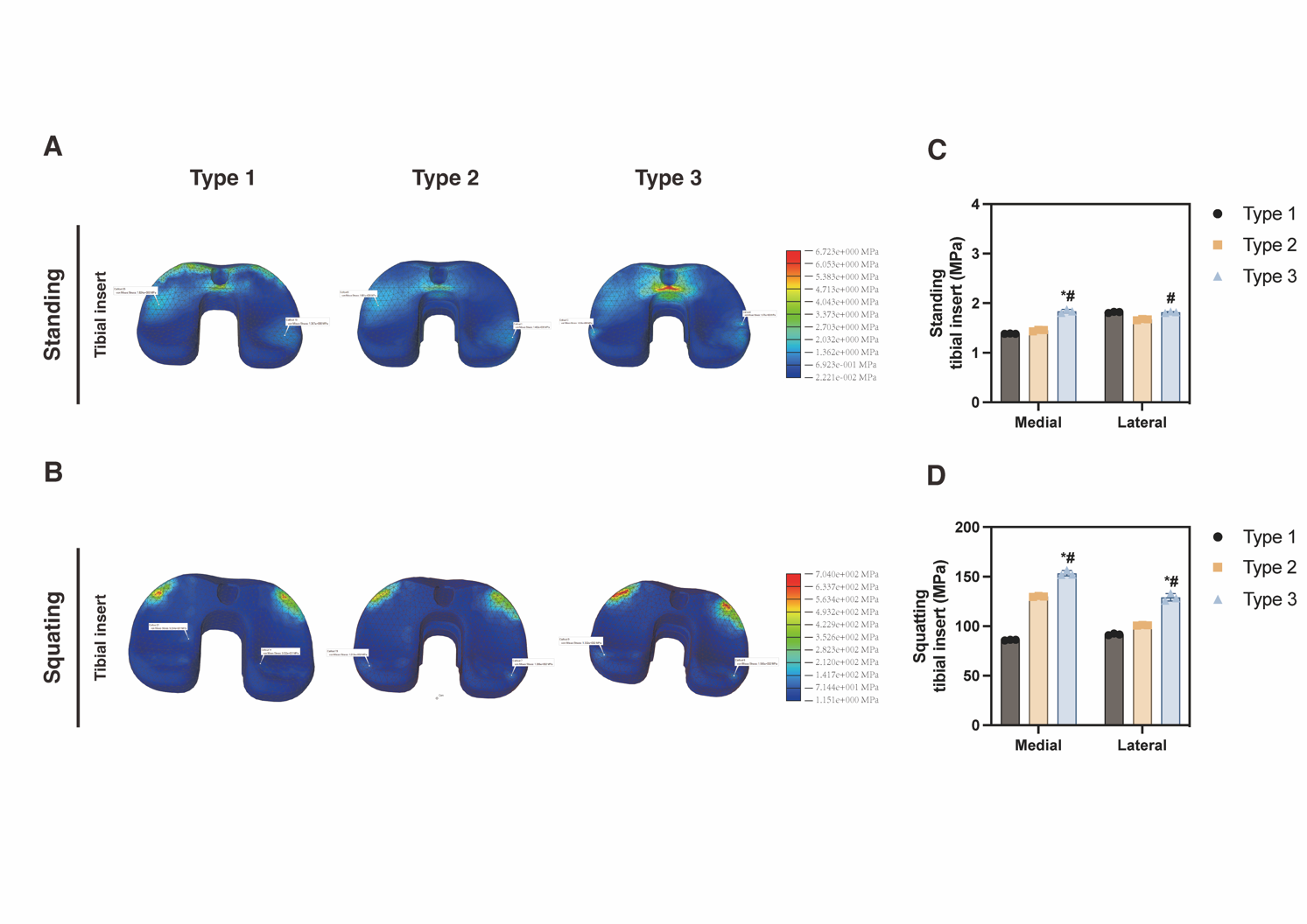


**Supplementary Figure 4.** (A,B) Stress distribution of the medial and lateral regions of the tibial inserts. The maximum von Mises stress within each region is pointed out. (C,D) Statistical analysis of the maximum von Mises stress in each region(**p* < 0.05 compared with Type 1, #*p* < 0.05 compared with Type 2).

**Supplement Table 1.** The maximum von Mises stress (MPa) and different rate (%) of Type 1 entire model at 700 N for different mesh densities.

| Mesh Density | Maximum von Mises stress (MPa) | Different Rate (%) |
| --- | --- | --- |
| 47009 | 63.93 | - |
| 58326 | 105 | 64% |
| 72579 | 74.83 | 29% |
| 98100 | 65.61 | 12% |
| 174264 | 52.56 | 20% |
| 192009 | 56.49 | 7% |
| 206142 | 81.15 | 44% |
| 276415 | 65.14 | 20% |
| 341411 | 82.95 | 27% |
| 496963 | 77.69 | 6% |
| 565925 | 75.22 | 3% |
| 904376 | 70.34 | 6% |
| 1239326 | 76.71 | 9% |

**Supplement Table 2.** The maximum von Mises stress (MPa) and different rate (%) of Type 3 entire model at 700 N for different mesh densities.

| Mesh Density | Maximum von Mises stress (MPa) | Different Rate (%) |
| --- | --- | --- |
| 44654 | 123.8 | - |
| 54818 | 134 | 8% |
| 67785 | 52.86 | 61% |
| 93946 | 39.7 | 25% |
| 134627 | 61.74 | 56% |
| 169211 | 32.01 | 48% |
| 201395 | 38.24 | 19% |
| 269000 | 33.39 | 13% |
| 333723 | 38.25 | 15% |
| 417284 | 37.01 | 3% |
| 646450 | 38.52 | 4% |
| 868185 | 39.36 | 2% |
| 1067964 | 36.51 | 7% |
